# Learning Full-Presence meditation using POEBRA: impacts on self-esteeem, benevolence, and anxiety

**DOI:** 10.3389/fpsyg.2026.1833806

**Published:** 2026-07-02

**Authors:** Anne Lieutaud, Helene Bourhis

**Affiliations:** Institute for Research, Innovation and Development (FP-I3ID)/Centre for Applied Research and Study on Perceptual Psychoeducation (CERAP), Fernando Pessoa University, Porto, Portugal

**Keywords:** embodied relationship to self, full-presence, interoception, learning from experience, meditation, self-compassion, self-esteem, trait anxiety

## Abstract

**Introduction:**

Meditation has shown benefits for mental health, yet difficulties may appear when practicing autonomously, particularly in people prone to anxiety or low self-esteem. The POEBRA (Program for Optimizing Self-Esteem and Benevolence and Reducing Anxiety) was developed to facilitate meditation learning through a structured, embodied, guided approach based on Full-Presence meditation. This study evaluated its effects on psychoaffective functioning in meditation novices, compared with an unguided silent practice.

**Method:**

The randomized, controlled, parallel-group study assigned 137 novice participants to either an eight-week POEBRA session (G1) or an unguided silence condition (G2). Participants completed five validated self-report scales before and after the intervention: Rosenberg's RSES, Neff's SCS, Spielberger's STAI_Y2, Crawford's PANAS, and Mehling's MAIA-2. Between-group differences in pre-post changes were analyzed using Welch's *t*-tests, complemented by ANCOVA and correlation analyses.

**Results:**

Completion rates were high (61/69 in G1 and 64/68 in G2). Compared with silence, POEBRA produced significantly greater improvements across all psychometric dimensions (all *p* < 0.001 except PANAS-NA, *p* = 0.015). The largest between-group effects were observed for trait anxiety (Δ = −8.1, *d* = 1.05), self-esteem (Δ = +3.6, *d* = 0.93), self-compassion (Δ = +0.53, *d* = 0.91), and interoceptive awareness (Δ = +0.54, *d* = 0.88). Positive affect increased under POEBRA (Δ = +4.1, *p* = 0.0002), while negative affect decreased more modestly. Correlation analyses revealed coherent internal dynamics in POEBRA, linking reduced anxiety with higher self-esteem and self-compassion (*r* = –0.64 and *r* = –0.68, both *p* < 0.001), while changes in the silence group were more diffuse.

**Discussion:**

Results confirm POEBRA strengthens self-esteem and self-compassion while reducing anxiety and emotional distress. Its emphasis on body awareness, guided reflection, and relational support appears to foster coherent psychoaffective transformation through embodied self-regulation. These findings align with previous research on the role of interoception and benevolence in emotional integration and self-confidence. Limitations include reliance on self-report measures and a predominantly female sample.

**Conclusion:**

POEBRA offers a structured, body-centered pedagogical framework that promotes emotional stability, benevolence, and self-confidence through Full-Presence meditation. By integrating sensory attention, reflective guidance, and relational coaching, it provides an accessible approach to cultivating embodied self-regulation in non-clinical populations.

## Introduction

Mindfulness-based meditation practices have gained widespread popularity in Western countries, extending beyond clinical settings to areas such as business and education. The scientific literature has extensively documented their positive impact on mental health.

Meditation is often perceived as a simple and accessible practice. Yet, in reality, many people find it difficult to meditate effectively. During meditation, individuals may encounter various challenges and even undesirable side effects (Britton et al., 2021), such as unpleasant sensations, fluctuating attention, frustration with unmet expectations, or negative self-evaluative thoughts. Achieving a state of accomplished meditation can be particularly challenging for those prone to anxiety or low self-esteem.

Research exploring the difficulties involved in learning meditation remains relatively scarce (Lindahl et al., 2017). Among the most recent contributions, Britton et al. (2021) identified a range of possible adverse experiences, including hyperstimulation, heightened anxiety, fear, insomnia, emotional instability, and either hypersensitivity or hyposensitivity. To mitigate such difficulties, several authors (Goleman and Davidson, 2017; Lutz, 2020) emphasize the importance of consistent practice under appropriate conditions, supported by sustained, guided training delivered by qualified professionals.

Empirical work on introspective, body-centered approaches—fostering a conscious relationship with bodily perception—has opened promising perspectives for addressing psychoaffective difficulties such as anxiety, low self-esteem, and reduced self-benevolence (Bois, 2018; Lieutaud et al., 2021; Lieutaud and Bois, 2018).

The *Program for the Optimization of Self-Esteem and Benevolence and the Reduction of Anxiety* (POEBRA) was developed in 2020 to facilitate access to meditation for individuals experiencing difficulties related to anxiety, self-esteem, or benevolence (Bois and Bourhis-Bois, 2026). POEBRA combines cognitive, metacognitive, and psychoaffective approaches to support personal transformation through bodily awareness. The program, based on Full-Presence meditation, a method derived from DBM-fasciatherapy, serves a dual purpose: first, to facilitate meditation learning by strengthening self-esteem and fostering benevolence toward oneself while alleviating anxiety; and second, to promote deeper personal transformation through the synergistic modulation of these three traits.

The three central constructs of POEBRA are operationalized as follows in the present study. Full-Presence designates a meditative practice derived from DBM-fasciatherapy (Bois and Eschalier, 2019) that places bodily and sensory perception—rather than attentional observation—at the center of the meditative process; rooted in Merleau-Ponty's phenomenology of perception, it conceives the body as the primary vector of self-knowledge and inner transformation. Benevolence is operationalized through Neff's Self-Compassion Scale (SCS), and more specifically its self-kindness subscale, which measures the disposition to treat oneself with warmth and understanding rather than self-criticism (Neff, 2003). Embodied self-regulation refers to the capacity to recognize and respond adaptively to internal bodily signals; it is captured by the Multidimensional Assessment of Interoceptive Awareness, version 2 (MAIA-2), whose dimensions of self-regulation, body listening, and trusting reflect the modulation of affective states through interoceptive attention (Mehling et al., 2018).

Although POEBRA shares with MBSR its 8-week structure—an intentional alignment to facilitate future comparative research—and its emphasis on non-judgmental awareness, it differs from established mindfulness-based interventions in three measurable respects. First, its primary therapeutic focus is the psychoaffective triad of self-esteem, benevolence, and anxiety, rather than stress or clinical symptoms as in MBSR and MBCT. Second, its pedagogical architecture follows a structured progression from the most factual to the most subjective, arranged across four levels: level 1 proposes factual exercises that are easy to carry out; level 2 emphasizes the relationship between feeling and subjectivity; level 3 invites participants to explore inner movement and changes in the internal environment; level 4 tests the ability to meditate without guidance and to integrate the week's key information. This progression is made possible by arranging verbal guidance so that instructions target, in sequence, procedural abilities, the cognitive sphere, the metacognitive sphere, and finally the psychoaffective sphere—a design absent from MBSR and MBCT. Third, its theoretical grounding rests on the primacy of sensory-perceptual experience—the body as the primary vector of self-knowledge—derived from DBM-fasciatherapy and Merleau-Ponty's phenomenology of perception, rather than on attentional monitoring or cognitive decentering models.

Our study aimed to evaluate, in meditation novices, the benefits of the POEBRA program compared with an unguided silent practice condition, on self-esteem, benevolence, and anxiety. This 2-arm randomized parallel study design was based on a quantitative longitudinal prospective evaluation method using five self-report psychometric tests to compare outcomes before and after the 8-week intervention.

## Material and methods

### Design and participants

The study followed a randomized controlled design with two parallel groups: an experimental group receiving the POEBRA intervention and an active comparison group engaged in autonomous unguided silent practice. Assessments were conducted at baseline (W0) and after 8 weeks (W8) using validated psychometric questionnaires.

This research was approved in July 2021 by the Ethics Committee of Fernando Pessoa University, PT. All participants signed an informed consent before starting the studied program.

Participants were recruited via mass mailing and social networks using an online questionnaire. The inclusion criteria were: be of legal age and French-speaking (our lab language within UFP); Have the desire to learn to meditate using a program focused on optimizing self-esteem, benevolence and reducing anxiety; Not have a regular practice of any form of meditation (less than 1 time per month); Not receive regular care (less than 1 time per month) by approaches having an assumed affiliation with Full-Presence meditation; Not begin, for the duration of the study, any form of physical or psychological practice. The online questionnaire also included questions regarding current psychological or psychiatric support and the use of psychotropic medication; none of the responses raised a concern incompatible with the study protocol. The screening relied on participant self-report and was not corroborated by a standardized clinical assessment.

Participants were randomly assigned to two study groups (G1 and G2) using a unique random number sequence generated with Excel's *RAND()* function. Within each group, participants were randomly divided into eight subgroups of equivalent size, each randomly assigned to the supervision of one of the eight instructors recruited for the study on a voluntary basis. Group allocation was disclosed at the kick-off meeting following baseline assessments. Due to the nature of the intervention and scheduling constraints, blinding and allocation concealment were not possible.

### The POEBRA program

The POEBRA program is an 8-week structured training designed to enhance self-awareness and emotional regulation through Full-Presence meditation (Bois and Bourhis-Bois, 2026). It combines daily guided meditations, body-centered perception exercises, and weekly reflective discussions led by certified facilitators trained in DBM-fasciatherapy, following a progressive pedagogical sequence targeting attention, interoception, and self-compassion. Each weekly session lasted 90 min. Between sessions, participants accessed an e-learning platform providing guided audio meditations, theoretical modules, and self-observation exercises to support their daily autonomous practice. This digital environment ensured content standardization, continuity of learning, and individualized follow-up. Facilitators were selected from POEBRA's international register of certified facilitators and received additional training to ensure protocol fidelity. Adherence and session attendance were monitored throughout the study to control for exposure to the intervention. An example of a Full-Presence guided meditation sequence is provided in Supplementary material S1 and on Pr. Danis Bois's personal page (Bois, 2018).

### Sample size

The trial was powered on the primary endpoint (change in STAI-Y2 from baseline to week 8): Assuming a moderate between-group standardized mean difference of *d* = 0.50 (two-sided α = 0.05, 80% power, 1:1 allocation), the required sample size was 63 participants per group (*N* = 126). Based on an anticipated attrition rate of 10%, the target sample size was set at 140 participants.

### Self-report psychometric tests

The evaluation focused on five self-reported psychometric scales: trait anxiety (STAI-Y2), self-esteem (RSES-10), self-compassion (SCS), affect balance (PANAS), and interoceptive body awareness (MAIA-2).

The primary outcome was the change in trait anxiety (STAI-Y2, W8–W0), reflecting the hypothesized reduction in anxious predisposition following the intervention. Changes in the remaining scales (RSES-10, SCS, PANAS, and MAIA-2) were defined as secondary outcomes. All psychometric instruments were administered to participants at baseline (W0) before randomization and after the 8-week intervention period (W8).

Trait anxiety was measured using Spielberger's State–Trait Anxiety Inventory (STAI, Y form) (Spielberger et al., 1983). The Trait-Anxiety scale (STAI-Y2) consists of 20 items rated on a 4-point Likert scale assessing the frequency of general feelings (1 = almost never, 2 = sometimes, 3 = often, and 4 = almost always). The scale yields a total score ranging from 20 to 80. A high score indicates the presence of high trait of anxiety.

Self-esteem was assessed using the Rosenberg Self-Esteem Scale (RSES) (Rosenberg, 1965). It is based on 10 items rating on a 4-point Likert scale self-appreciation with some implicit comparison of oneself to others. The scale yields a single total score ranging from 10 to 40, with higher scores indicating more positive self-esteem.

Self-compassion, based solely on a relationship of benevolence toward oneself, was evaluated with Neff's Self-Compassion Scale (SCS) (Neff, 2003), comprising 26 items rated on a 5-point Likert scale (*almost never* to *almost always*). It assesses three bipolar dimensions of self-compassion (6 subscales). The total self-compassion score is the grand mean of all six-subscale means, and ranges between 1 and 5, with a higher score indicating more self-compassion.

Emotional mood tendencies were assessed using the Positive and Negative Affect Schedule (PANAS) (Watson et al., 1988). It includes 20 adjectives (10 positive, 10 negative) rated on a 5-point scale (*very slightly or not at all* to *extremely*). The PANAS score is separated into the Positive Affect (PA) and Negative Affect (NA) scores, each ranging from 10 to 50, with a higher score indicating higher trend for positive or negative affect, respectively.

Interoceptive awareness was assessed using the Multidimensional Assessment of Interoceptive Awareness, version 2 (MAIA-2) (Mehling et al., 2018), a 37-item self-report scale rated on a 6-point Likert scale (0 = *never* to 5 = *always*). The eight-factor structure captures dimensions of body awareness, attention, and confidence. At the time of study design, no validated French translation of the MAIA-2 was available. The French working translation available on Mehling's institutional website was found to contain several significant mistranslations; these were systematically corrected according to the theoretical framework of each subscale, with the confirmative input of a native English somatic-psychoeducator. Subsequent to data collection, Da Costa Silva et al. (2022) published a validated French adaptation of the MAIA-2 in a non-clinical French-speaking adult sample. Alignment was verified by item-by-item comparison; the majority of items were identical or showed only minor lexical variants, and the remainder used semantically equivalent phrasing within the framework of each subscale. Following the recommendation of Da Costa Silva et al. (2022) an overall score was computed as the mean of six of the eight factors, ranging from 0 to 5. Higher scores equate to more awareness of bodily sensation. Internal consistency was excellent across factors (Cronbach's α = 0.77–0.88).

### Statistical analyses

Only quantitative data were collected and analyzed. Over 20 000 entries were organized into 3 files (sociodemographics, weekly diaries and psychometrics), were evaluated and cleaned according to the recommendations of Chin and Lee (2008): data entry errors were corrected when the error mechanism could be decomposed (36 outliers corrected on the basis of weekly experience diaries, 18 corrections of systematic errors due to an artifact of the online questionnaire administration instrument,...); one irreplaceable item of data (a technical incident during the recording of data entry) and important for the dataset (1 of the 5 self-report scales at W0 of one person who actually completed the entire course) was substituted by the mean value of people with the same socio-demographic characteristics, following Chin and Lee's “Hot Deck Imputation” principle (*Ibid*).

Within-group W8–W0 changes on the six psychometric outcomes were assessed using Welch's paired *t*-tests, and pairwise associations between these changes within each group were examined using Spearman's rank correlation coefficient (ρ). Both tests were preferred to their parametric alternatives for their robustness to outliers and to potential non-normality of the W8–W0 difference scores. Between-group contrasts on the same outcomes were estimated by analysis of covariance (ANCOVA) modeling (W8–W0) ~ W0 + group, with the group effect reported as the estimated marginal mean (EMM) difference and its 95% confidence interval (Van Breukelen, 2006; Vickers and Altman, 2001). The multivariate structure underlying the correlations was further explored using principal component analysis (PCA) (Supplementary material S2). The Holm-Bonferroni step-down correction (Holm, 1979) was applied within each family of related tests at α = 0.05 family-wise. The robustness of the between-group effect to age adjustment was verified by sensitivity analyses (Supplementary material S3).

Effect sizes were calculated using Cohen's *d*, defined as the mean difference divided by the pooled standard deviation. According to conventional thresholds, values of 0.2, 0.5, 0.8, and above 1.0 were interpreted, respectively as small, medium, large, and very large effects (Cohen, 1988). Spearman correlations were interpreted as weak (|ρ| <0.30), moderate (0.30 ≤ |ρ| <0.50), or strong (|ρ| ≥ 0.50).

The Minimal Clinically Important Difference (MCID) was estimated for each psychometric variable to complement the statistical significance analyses. MCID thresholds were defined as 0.5 × the baseline standard deviation of each scale, following the distribution-based method recommended for psychological measures. Participants whose individual change (Δ W8–W0) exceeded this threshold were considered to have achieved a clinically meaningful improvement. The proportion of responders per group was then compared using χ^2^ tests, and the resulting class distributions are graphically visualized.

All analyses were performed using R (v4.1.0), with two-tailed significance set at *p* < 0.05. Estimated marginal means and confidence intervals were computed with the emmeans package; principal component analysis with FactoMineR and factoextra; partial Spearman correlations with ppcor. Graphical representations (boxplots, correlation matrices, PCA biplots) were generated using the ggplot2 and FactoMineR packages. The anonymized dataset and the R scripts used for the statistical analyses are openly accessible for research and verification purposes, in compliance with ethical and data protection standards (see Data Availability Statement).

## Results

The aim of the study was to measure the effects of POEBRA on issues of anxiety, self-esteem, and self-compassion in non-clinical meditation novices and investigate the possible benefits from interoception when used as a learning support.

### Recruitment

**Figure 1:**
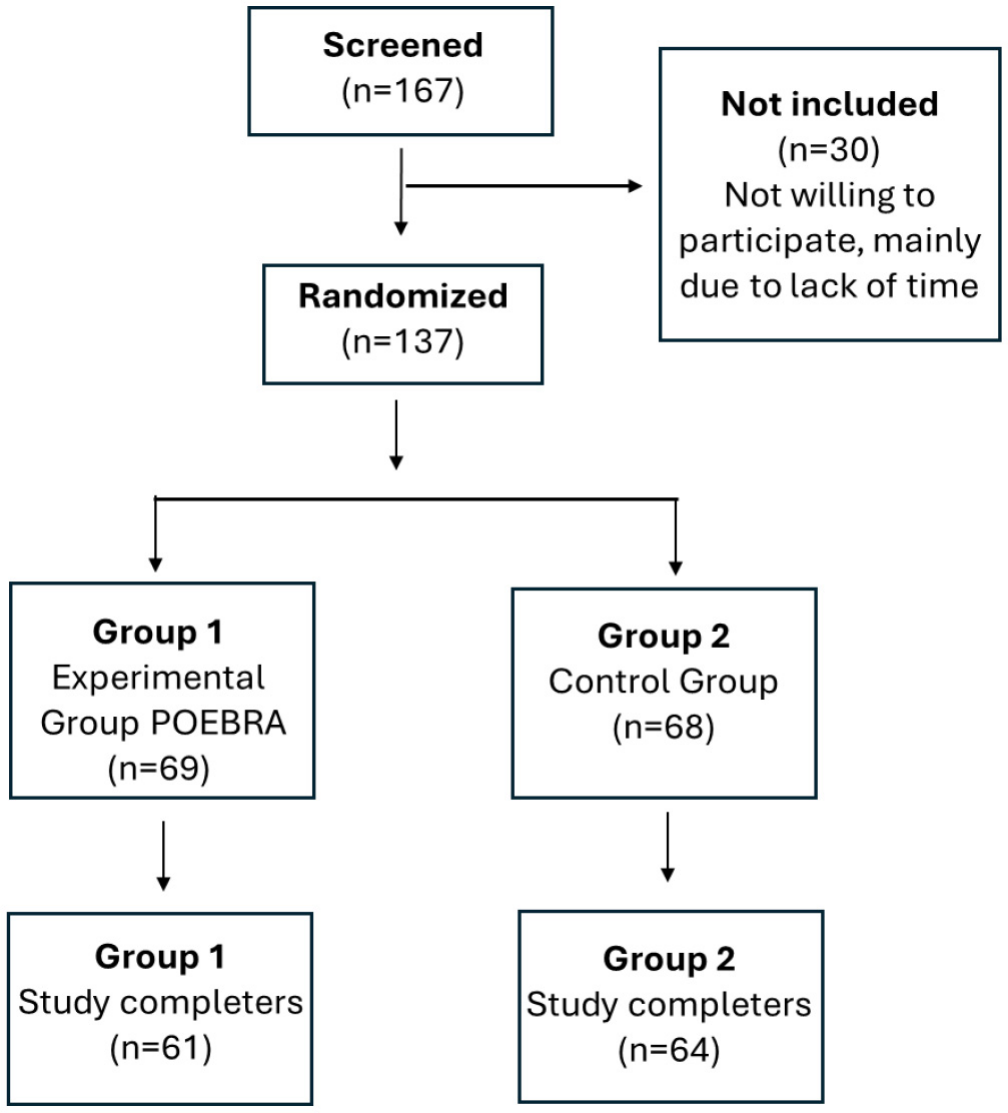
Flow diagram of participant selection, randomization, and study completion. A total of 137 participants were randomized into two groups: the POEBRA experimental group (*n* = 69) and the control group practicing silent meditation in autonomy (*n* = 68). After 8 weeks of intervention, 61 participants completed the study in the POEBRA group and 64 in the control group (*N* = 125). Reasons for non-inclusion and attrition are indicated where applicable.

The study took place between January and March 2022. A total of 137 participants were randomized after screening (cf Figure 1) following intention-to-treat principles. Sixty-nine were allocated to the experimental group (POEBRA) and sixty-eight to the control group. The G2 group, subjected to simple non-mediated listening to silence, acted as an active control group for the G1 intervention group.

At study completion, 61 participants in the experimental group and 64 in the control group had completed all study assessments. Reasons for non-inclusion are detailed where applicable. In group 1, attrition over the 8 weeks was divided roughly equally between people who had difficulties with the time and commitment required and people who simply stopped participating (lost to sight). And in group 2, attrition consisted of dropouts and lost of sight.

### Socio-demographic data

The majority of participants were women (*n* = 110, 80%) (cf Table 1). The average age of the participants was 46, 7 years, SD ±11, 1 years, [20–70]. The distribution of age was not different between both groups.

**Table 1:** Socio-demographic characteristics of participants.

Characteristics	Sub-characteristics	Total sample (*n* = 137)		Group G1 (*n* = 69)		Group G2 (*n* = 68)	
		Proportion (number)	Mean ±SD	Proportion (number)	Mean ±SD	Proportion (number)	Mean ±SD
Gender	Male	19.7% (*n* = 27)		14.5% (*n* = 10)	44.0 years ±9.8	25% (*n* = 17)	45.1 years ±12.2
	Female	80.3% (*n* = 110)		85.5% (*n* = 59)	47.2 years ±10.6	75% (*n* = 51)	46.3 years 11.8
	Other					*n* = 1	23.0 years
Age			46.4 years ±11.1		46.8 years ±10.5		46.0 years ±11.8
Nber of years of education			15.4 ± 2.0		15.3 ± 2.3		15.4 ± 2.4
Education level	No higher education	18.2% (*n* = 25)	11.7 ± 0.8	20.3% (*n* = 14)	11.9 ± 0.5	16.2% (*n* = 11)	11.5 ± 0.9
	2 years of higher education	34.3% (*n* = 47)	14.5	30.4% (*n* = 21)	14.5	38.2% (*n* = 26)	14.5
	Master's deg. or higher	47.4% (*n* = 65)	17.4 ± 1.1	49.3% (*n* = 34)	17.3 ± 1.0	45.6% (*n* = 31)	17.6 ± 1.2
Marital status	In a relationship	57.7% (*n* = 79)		58.0% (*n* = 40)		57.4% (*n* = 39)	
	Single	28.5% (*n* = 39)		24.6% (*n* = 17)		32.4% (*n* = 22)	
	Single parent	13.9% (*n* = 19)		17.4% (*n* = 12)		10.3% (*n* = 7)	

### Changes in main psychometric scores

Inferential analyses were organized into four pre-specified families of tests addressing distinct research questions: (F1) within-group W8–W0 changes in G1; (F2) within-group W8–W0 changes in G2; (F3) between-group ANCOVA contrasts on (W8–W0), modeled as (W8–W0) ~ W0 + group, with the group effect reported as the estimated marginal mean (EMM) difference and its 95% confidence interval; and (F4) Spearman rank-order correlations between W8–W0 changes for the 15 unique outcome pairs, computed within each group. The Holm-Bonferroni step-down correction was applied within each family at α = 0.05 family-wise. A complete table of raw and Holm-adjusted *p-values* for the four families is reported in Supplementary material S4. To verify that the primary group effect on STAI-Y2—and the corresponding effects on the five secondary outcomes—are not confounded by age, three complementary sensitivity analyses were performed (see Supplementary material S3): an age-adjusted ANCOVA, an exploratory group × age interaction test, and partial Spearman correlations between W8–W0 changes and age while controlling for W0 and group.

Across the six main outcomes (two for the PANAS questionnaire), POEBRA (G1, *n* = 61) consistently shows larger and more significant improvements than silent practice (G2, *n* = 64) after Holm-Bonferroni correction (cf Table 2, F1, F2, and F3 rows named as “within” for G1 and G2, and as “ANCOVA EMM” for the between-group difference):

- Trait Anxiety (STAI-Y2): Anxiety markedly decreases in G1 (Δ = –*10.46*, SD = 8.45, Cohen's *d* = −1.24), compared to a mild reduction in G2 (Δ = –*2.34*, SD = 6.90, *d* = −0.34). The between-group contrast [Δ = −8.07 (−10.66; −5.48), *d* = −1.05, *p* < 0.001] yields a very large effect size, confirming a robust anxiolytic impact of POEBRA.
- Self-Esteem (RSES): Self-esteem significantly increases in G1 (Δ = +*4.41*, SD = 4.22, *d* = +1.05), while G2 shows no significant change (Δ = +*0.84*). The between-group difference [Δ = *3*.37 (+2.06; +4.67), *d* = +0.88, *p* < 0.001] denotes a strong effect, consistent with greater self-valuation under POEBRA. However, a marginal violation observed for RSES (W0 × group interaction *p* = 0.044) should moderate this interpretation.
- Self-Compassion (SCS): G1 participants show a strong increase in self-compassion (Δ = +*0.78*, SD = 0.60; *d* = +1.30), compared with a modest gain in G2 (Δ = +*0.26*, SD = 0.55; *d* = +0.47). The between-group contrast [Δ = *0.54* (+0.36; +0.72), *d* = +0.91, *p* < 0.001] indicates a large effect size, confirming that POEBRA substantially enhances self-compassion.
- Body Awareness (MAIA-2): The improvement in interoceptive awareness is also higher in G1 (Δ = +*0.89*, SD = 0.65; *d* = +1.36) than in G2 (Δ = +*0.35*, SD = 0.64; *d* = +0.55). The between-group estimated difference [Δ = +0.66 (+0.45; +0.88), *d* = +0.83, *p* < 0.001] reflects another strong effect, suggesting that POEBRA reinforces body–mind connection more effectively. However, a violation was observed on the HOV assumption (W0 × group interaction *p* = 0.007), which calls for caution on the precise EMM value but does not affect the direction of the effect: the within-group magnitudes themselves (G1: *d* = +1.36 vs. G2: *d* = +0.55, both Holm-significant) confirm a substantially larger improvement in G1.
- Positive Affect (PANAS-PA): G1 shows a marked increase in positive affect (Δ = +*4.46*, SD = 5.64; *d* = +0.79), while G2 remains stable (Δ = +*0.34, ns*). The between-group difference [Δ = *4*.11 (+2.36; +5.86), *d* = +0.69, *p* < 0.001] corresponds to a moderate-to-strong effect, indicating a clear improvement in emotional wellbeing under POEBRA.
- Negative Affect (PANAS-NA): Both groups reduce negative affect, but more strongly in G1 (Δ = –*7.00*, SD = 7.30; *d* = −0.96) than in G2 (Δ = –*3.84*, SD = 6.94; *d* = −0.55). The between-group difference [Δ = −3.33 (−5.40; −1.26) (*d* = −0.44, *p* = 0.002)] represents a moderate effect, confirming a more pronounced emotional relief with POEBRA.

**Table 2:** Psychometric outcomes: within-group changes (G1, G2) and between-group ANCOVA contrasts (W8–W0).

Outcome	Comparison	*n*	M (SD) at W0	M (SD) at W8	Effect [Δ M (SD) within/EMM (95% CI) between]	Cohen's *d*	*p* (Holm)
STAI-Y2 (trait anxiety, primary)	G1 (within)	61	52.20 (10.90)	41.90 (10.70)	−10.46 (8.45)	−1.24	<0.001
	G2 (within)	64	51.90 (10.30)	49.80 (11.10)	−2.34 (6.90)	−0.34	0.025
	G1–G2 (ANCOVA EMM)	125	—	—	−8.07 [−10.66; −5.48]	−1.05	<0.001
RSES (self-esteem, secondary)	G1 (within)	61	27.30 (6.14)	31.80 (5.00)	+4.41 (4.22)	+1.05	<0.001
	G2 (within)	64	28.50 (5.57)	29.00 (5.90)	+0.84 (3.87)	+0.22	0.172
	G1–G2 (ANCOVA EMM)	125	—	—	+3.37 [+2.06; +4.67]^†^	+0.88	<0.001
SCS (self-compassion, secondary)	G1 (within)	61	2.54 (0.70)	3.34 (0.70)	+0.78 (0.60)	+1.30	<0.001
	G2 (within)	64	2.54 (0.62)	2.78 (0.60)	+0.26 (0.55)	+0.47	0.001
	G1–G2 (ANCOVA EMM)	125	—	—	+0.54 [+0.36; +0.72]	+0.91	<0.001
MAIA-2 (interoception, secondary)	G1 (within)	61	2.56 (0.93)	3.44 (0.72)	+0.89 (0.65)	+1.36	<0.001
	G2 (within)	64	2.15 (0.79)	2.49 (0.95)	+0.35 (0.64)	+0.55	<0.001
	G1–G2 (ANCOVA EMM)	125	—	—	+0.66 [+0.45; +0.88]^‡^	+0.83	<0.001
PANAS-PA (positive affect, secondary)	G1 (within)	61	29.50 (5.97)	34.20 (5.33)	+4.46 (5.64)	+0.79	<0.001
	G2 (within)	64	29.90 (6.62)	30.10 (6.15)	+0.34 (6.19)	+0.06	0.658
	G1–G2 (ANCOVA EMM)	125	—	—	+4.11 [+2.36; +5.86]	+0.69	<0.001
PANAS-NA (negative affect, secondary)	G1 (within)	61	26.30 (7.82)	19.40 (6.51)	−7.00 (7.30)	−0.96	<0.001
	G2 (within)	64	26.80 (8.12)	22.90 (7.40)	−3.84 (6.94)	−0.55	<0.001
	G1–G2 (ANCOVA EMM)	125	—	—	−3.33 [−5.40; −1.26]	−0.44	0.002

G1 (*n* = 61) and G2 (*n* = 64) refer to the two participant groups as defined in the Methods section. W0 = baseline (week 0, before the 8-week programme); W8 = post-intervention (week 8). The trial reports one primary outcome (STAI-Y2 trait anxiety) and five secondary outcomes (RSES, SCS, MAIA-2, PANAS-PA, PANAS-NA). Instruments and scoring direction (higher = more of the construct): STAI-Y2, State-Trait Anxiety Inventory, trait subscale; RSES, Rosenberg Self-Esteem Scale; SCS, Self-Compassion Scale, total score; MAIA-2, Multidimensional Assessment of Interoceptive Awareness, version 2, total score (computed as the mean of 6 of the 8 subscales; see Methods); PANAS-PA / PANAS-NA, Positive Affect / Negative Affect Schedule. Scoring ranges and directions of all instruments are defined in Methods.

Column definitions. M (SD) at W0 / at W8 = group mean and standard deviation of the raw score at each time point. Effect = raw effect on the instrument's own scale: for the within-group rows (G1, G2), it is the mean change Δ M (SD) = mean of (W8–W0) with its standard deviation across participants; for the between-group row (G1–G2 ANCOVA EMM), it is the estimated marginal mean difference between groups in the W8–W0 change after adjustment for the W0 baseline, with its 95% confidence interval. Cohen's d = standardized effect size; interpretation conventions are defined in Methods.

*p* (Holm) = Holm-Bonferroni adjusted p-value within each family of six tests (within-G1, within-G2, between-groups); statistical methods are described in detail in Methods. Values shown as <0.001 are below 10^−^.

^†^Marginal violation of the homogeneity-of-slopes assumption underlying ANCOVA (W0 × group interaction *p* < 0.05): RSES (*p* = 0.044).

^‡^More pronounced violation: MAIA-2 (*p* = 0.007). See Discussion (Robustness of the ANCOVA estimates) and Supplementary material S3 for sensitivity analyses confirming the direction and significance of these two effects.

The two violations of the homogeneity-of-slopes assumption underlying ANCOVA (W0 × group interaction *p* < 0.05) led us to look for confounding factors. But the age-adjusted sensitivity analyses, detailed in Supplementary material S3, yielded virtually identical EMM differences and confirmed Holm-significance on all six outcomes. We therefore consider the suggested interpretation to be sufficiently robust, although further confirmation studies would be warranted.

### Correlations between changes in psychometric variables (W8–W0)

The Spearman correlations between W8–W0 changes (Table 3) reveal three notable patterns across the two groups:

- The strongest association in both groups is between changes in self-compassion and trait-anxiety reduction (SCS ↔ STAI-Y2: ρ = −0.68 in G1, −0.64 in G2), making this link the dominant coupling axis of the psychoaffective transformation regardless of practice condition.
- Changes in interoceptive awareness (MAIA-2) are only weakly associated with the other outcomes: in G1, none of the MAIA-2 correlations reach Holm-adjusted significance, while in G2 only the SCS ↔ MAIA-2 link does (ρ = +0.35).
- The coupling between negative-affect change (PANAS-NA) and self-related changes is markedly stronger in G2 than in G1—particularly with self-compassion (ρ = −0.53 in G2 vs. −0.29 NS in G1) and self-esteem (ρ = −0.37 in G2 vs. −0.35 NS in G1).

**Table 3:** Spearman rank-order correlations between W8–W0 changes for the primary outcome (STAI-Y2) and the five secondary outcomes (G1 below the diagonal; G2 above the diagonal).

Outcomes	STAI-Y2	RSES	SCS	MAIA-2	PANAS-PA	PANAS-NA
STAI-Y2	—	−0.482^*^	−0.644^*^	−0.282	−0.344^*^	0.567^*^
RSES	−0.643^*^	—	0.360^*^	0.192	0.166	−0.374^*^
SCS	−0.680^*^	0.560^*^	—	0.351^*^	0.385^*^	−0.528^*^
MAIA-2	−0.321	0.220	0.229	—	0.316	−0.187
PANAS-PA	−0.520^*^	0.263	0.339	0.222	—	−0.313
PANAS-NA	0.366^*^	−0.351	−0.286	0.088	−0.348	—

See Table 2 for outcome abbreviations. Values below the diagonal correspond to group G1 (*n* = 61); values above the diagonal correspond to group G2 (*n* = 64). The diagonal is left empty (—). An asterisk (^*^) marks correlations significant after Holm-Bonferroni correction within each group across the 15 outcome pairs (α = 0.05 family-wise). Raw and Holm-adjusted p-values for all 30 correlations are reported in Supplementary material S4. Magnitude interpretation conventions: |ρ| = 0.10 = small, = 0.30 = medium, ≥0.50 = large.

The two latter patterns—partial independence of interoception and selective decoupling of negative affect under POEBRA—raise the question of whether the latent organization of psychoaffective change differs between the two groups, beyond a simple difference in the magnitude of bivariate associations. Raw and Holm-adjusted *p-values* for all 30 correlations are reported in Supplementary material S4.

### Multivariate analysis of changes (PCA)

**Figure 2:**
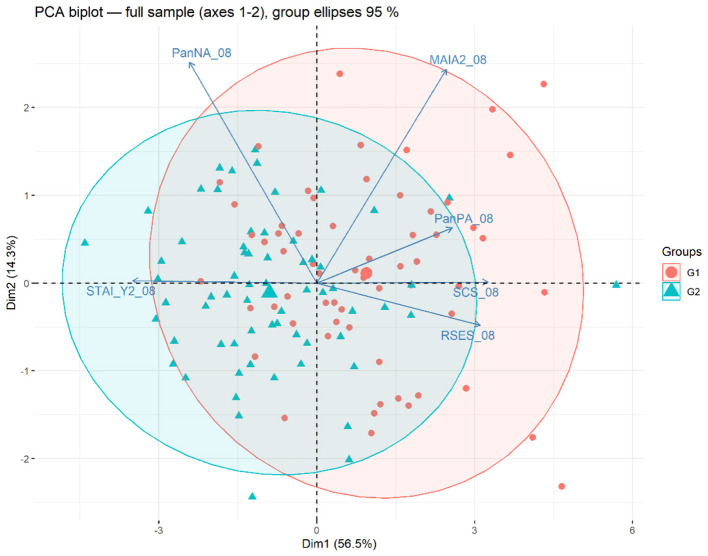
PCA biplot of W8–W0 changes—full sample (*n* = 125), axes 1–2, with 95% confidence ellipses by group.

To address this question, a principal component analysis (PCA) was performed on the W8–W0 changes of the six outcomes, both on the full sample (*n* = 125, with the group factor projected as a supplementary qualitative variable) and within each group separately (Supplementary material S2). The first axis captures a unified direction of clinical benefit on which all six outcomes load coherently, accounting for the largest share of the variance (56.5% in the full sample; 49.3% in G1; 52.7% in G2). G1 participants are projected substantially further along this benefit-aligned axis than G2 participants, with limited overlap between the 95% group ellipses (Figure 2), in concordance with the ANCOVA contrasts. Beyond this shared first axis, the latent organizations of the two groups differ informatively. In G1, the second axis (18.5% of the variance) groups changes in interoceptive awareness with changes in negative affect (MAIA-2 contributing 57.6% to the axis; PANAS-NA, 37.1%), articulating an introspective-somatic dimension. In G2, the second component (14.3%) is dominated by changes in positive affect (PANAS-PA: 74.8% of the axis), with a markedly heterogeneous distribution—the coefficient of variation of the W8–W0 change in PANAS-PA is approximately 14 times higher in G2 than in G1; the introspective-somatic dimension is relegated to the third axis in G2 (MAIA-2: 59.7%; PANAS-NA: 35.3%). Eigenvalues, loadings, contributions and squared cosines for the three analyses are reported in Supplementary material S2.

### Age-sensitivity analyses

To verify that the between-group effects reported above are not confounded by participants' age, three sensitivity analyses were conducted on the same sample (Supplementary material S3). First, the primary ANCOVA was re-estimated with age added as an additional covariate; the age-adjusted EMM differences deviated from the primary estimates by at most a 100th of a unit on each scale and remained Holm-significant on all six outcomes. Second, an exploratory group × age interaction term was tested for each outcome; none of the six tests approached significance (all *p* > 0.49). Third, partial Spearman correlations between W8–W0 changes and age, controlling for W0 and group, were small in magnitude (|ρ| ≤ 0.18) and none reached significance at α = 0.05 (smallest *p* = 0.057, for SCS). Taken together, the three analyses indicate that the magnitude of the between-group effect on the primary and secondary outcomes is homogeneous across the age range of the sample and is not modified by adjustment for age.

Taken together, these inferential results indicate (i) a coherent multivariate transformation along a unified benefit dimension across all six outcomes, substantially more pronounced in G1 than in G2; (ii) a self-compassion ↔ trait-anxiety axis as the dominant psychoaffective coupling regardless of practice condition; (iii) an introspective-somatic dynamic involving interoceptive awareness and negative affect that occupies a structurally central position in G1 but is relegated to a secondary axis under autonomous practice; (iv) a decoupling of negative-affect change from self-related changes specific to G1, suggesting a distinctive reorganization of the psychoaffective architecture under guided practice; and (v) homogeneity of the between-group effect across the 20–70 age range of the sample, supporting the generalizability of these findings to a broad adult audience.

### Clinically meaningful changes (MCID)

**Figure 3:**
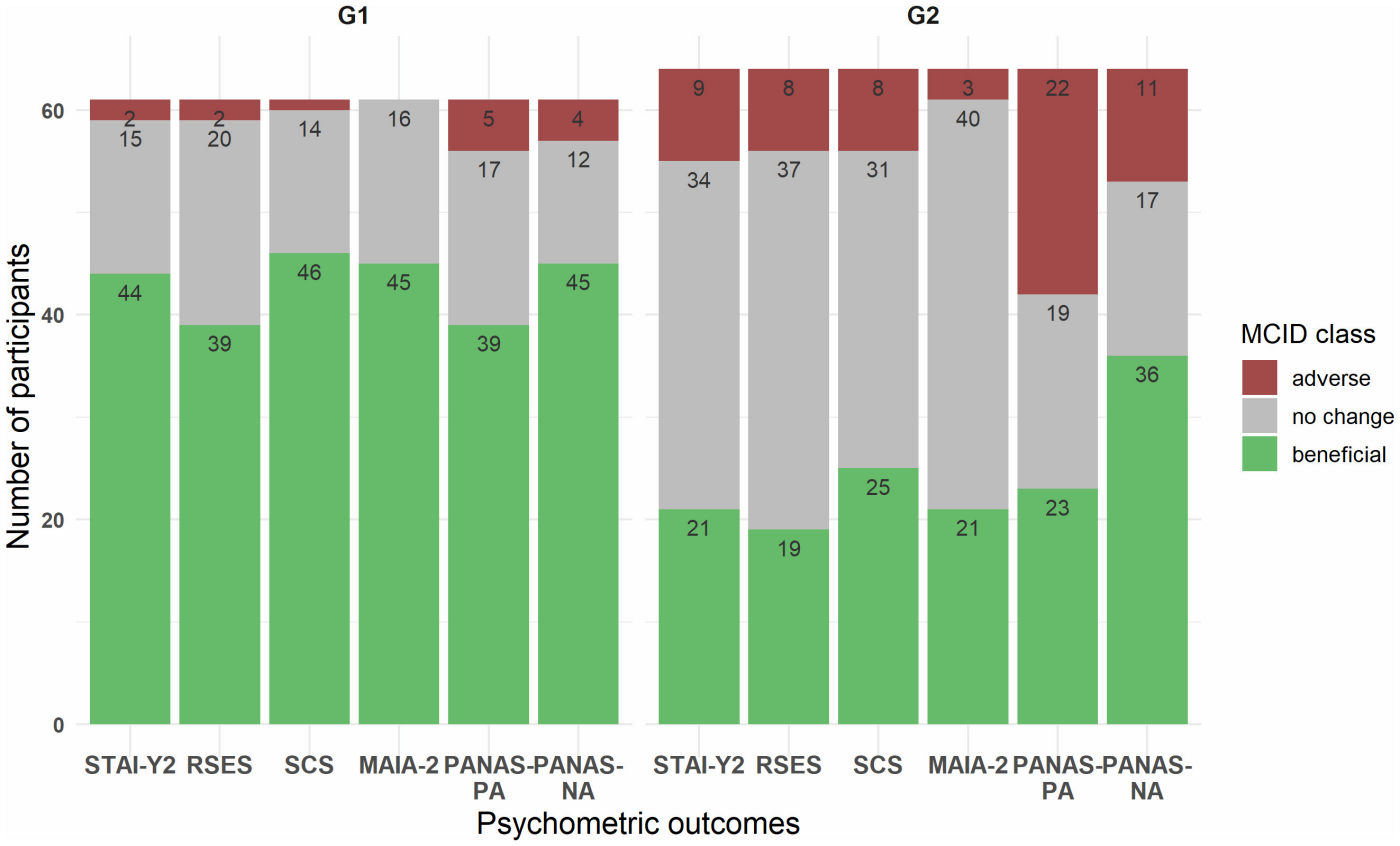
Categories of psychometric changes by group. Changes are classified as “pos” for beneficial, “neg” for negative or “null” when there is no significant change (with inversion for STAI-Y2 and PANAS-NA). Category totals are shown in each segment of the chart bars.

Beyond statistical significance, the analysis also explored whether the magnitude of change observed on each psychometric scale corresponded to a clinically meaningful improvement for participants, based on the Minimal Clinically Important Difference (MCID). Figure 3 summarizes the distribution of MCID across the six psychometric variables in both groups. Changes were classified as *positive, negative*, or *null* according to the predefined MCID (0.5 × SD at W0) for each scale. For STAI-Y2 and PANAS-NA, the direction of change was reversed so that “positive” consistently represents clinical improvement.

The figure shows a clear advantage of POEBRA (G1) over silent practice (G2). The proportion of participants showing clinically significant improvement is nearly twice as high in G1 across most variables, except for negative affect (PANAS-NA), which improves in both groups. Conversely, G2 includes a much higher proportion of individuals with *no significant change*, particularly on the STAI-Y2, SCS, RSES, and MAIA-2 scales.

Overall, POEBRA appears to promote a more homogeneous and consistently beneficial response profile. Only one participant in G1 showed no positive change, compared with 13 in G2, while 15 participants in G1 improved across all variables (vs. only 4 in G2). Negative patterns were rare and almost exclusively found in G2.

## Discussion

The present study demonstrates that participation in the POEBRA program, an 8-week structured training in Full-Presence meditation, produces consistent and significant improvements in key psychoaffective domains among meditation novices. Compared with autonomous silent practice, POEBRA resulted in greater reductions in trait anxiety and greater increases in self-compassion, self-esteem, positive affect, and interoceptive awareness and the between-group contrast reached a large to very large effect size on all outcomes except negative affect (moderate effect size). The magnitudes of these improvements are not only statistically significant but clinically meaningful, as the gains across all six outcomes in the POEBRA group (G1) exceeded the Minimal Clinically Important Difference threshold, in a substantially higher proportion than in the silence group (G2). These findings demonstrate the transformative reach of the program as an integrated design and confirm the relevance of an embodied and guided pedagogical framework for fostering sustainable psychological transformation.

The magnitude of the effect sizes reported here are situated within the range of values reported in the meditation literature. Within-group pre-post Cohen's *d* values for G1 participants range from 0.79 to 1.36 across the six outcomes, reflecting, in large part, the profile of our sample: motivated, self-selected, meditation-naive adults with substantial room for improvement at baseline—a configuration that consistently yields large within-group effects in practitioner-designed non-clinical programs (for comparable values in the Mindful Self-Compassion program, see Neff and Germer, 2013). The methodologically appropriate benchmark for evaluating POEBRA's specific added value is, however, the between-group ANCOVA-estimated contrast, which represents the advantage of G1 over G2 adjusted for baseline differences. These between-group contrasts (*d* = 0.44 to *d* = 1.05) fall within, sometimes slightly above, the ranges reported in 8-week mindfulness-based intervention RCTs: *d* = 0.4–0.7 for anxiety outcomes (Hofmann et al., 2010; Khoury et al., 2015), *d* = 0.6–1.1 for self-compassion-targeted programs (Neff and Germer, 2013), and *d* = 0.3–0.75 for self-esteem and positive affect outcomes in reviews of MBI psychological effects (Keng et al., 2011; Randal et al., 2015; Sedlmeier et al., 2012). Where our contrasts reach the upper end of these benchmarks, this reflects the nature of the comparison design—structured guided practice vs. autonomous unguided silent practice capturing the full added value of structured pedagogical scaffolding, its specific meditative content and the non-specific contextual factors of structured participation, as acknowledged in the Limitations section.

The strongest psychoaffective coupling observed in the present sample—between changes in self-compassion and trait anxiety, with ρ = −0.65 to −0.68 across both groups—is consistent with one of the most robust findings in the self-compassion literature: a strong inverse relationship between self-compassion and anxiety, replicated across populations and intervention types (MacBeth and Gumley, 2012; Neff, 2003; Neff and Vonk, 2009). Notably, this coupling holds in both groups regardless of the practice, suggesting that even autonomous silence practice naturally cultivates a benevolent attitude toward oneself which, in turn, appears to support the reduction of anxious appraisal. This is consistent with conceptual proposals that locate self-compassion as one of the central mechanisms of action of contemplative practices (Hölzel et al., 2011; Neff and Germer, 2013), and with previous work highlighting the role of embodied awareness in the development of self-trust and affective resilience (Bourhis et al., 2017; Lieutaud and Bois, 2018). The specific contribution of POEBRA likely resides in the markedly larger magnitude of self-compassion gain achieved through the integration of body-centered attention and reflective guidance, which together promote a more stable and embodied form of self-relation than autonomous practice alone.

The position of interoceptive awareness in the present findings deserves a specific discussion. Beyond a simple perception of the body, the MAIA-2 instrument captures a multidimensional relationship to the lived body—an embodied self-experience that includes attention to sensations, emotional awareness, and the cultivation of the body as a trusted ally in self-regulation (Mehling et al., 2012, 2018). In the principal component analysis (Supplementary material S2), the first axis captures the direction of clinical benefit on which all six outcomes align, while the second captures which dimension carries the greatest structural weight in explaining individual variation, after this main orientation. In G1 (POEBRA participants), the second axis is defined by changes in interoceptive awareness (MAIA-2: 57.6% of the axis) and negative affect (PANAS-NA: 37.1%): the embodied affective register stands as the most structurally central dimension. In G2, by contrast, this dimension is relegated to the third axis, displaced by changes in positive affect (PANAS-PA: 74.8%, with markedly heterogeneous distribution). Pedagogical scaffolding therefore does not change the direction of improvement but reorganizes the structural importance of the dimensions through which it is articulated: with structured guidance, embodied self-awareness becomes structurally central, woven with affective regulation; without it, the heterogeneity of mood responses dominates. This articulation is consistent with models locating embodied awareness at the core of self-related and emotional processing (Bechara and Naqvi, 2004; Damasio, 2021; Domschke et al., 2010; Farb et al., 2015), with research identifying interoception as foundational to mental health (Khalsa et al., 2018), and with empirical work tying embodied awareness specifically to emotional regulation (Khoury et al., 2017; Price and Hooven, 2018; Schmalzl et al., 2014).

We wish to stress another distinctive multivariate finding: the asymmetry in the coupling between negative-affect change and self-related changes: in G2, changes in negative affect are tightly coupled to changes in self-compassion (ρ = −0.53), self-esteem (ρ = −0.37), and trait anxiety (ρ = +0.57); in G1, this coupling is markedly attenuated (ρ between −0.29 and +0.37, all non-significant or marginal after Holm correction). This asymmetry suggests that POEBRA cultivates not only a larger magnitude of psychoaffective change but also a reorganization of the dependencies between dimensions of inner experience. Specifically, the dampening of the link between mood (negative affect) and the cognitive-evaluative dimension (anxiety, self-compassion, self-esteem) parallels what the contemplative literature describes as decentering or metacognitive awareness: the capacity to observe one's affective and cognitive states as transient experiences rather than as truths about the self (Bernstein et al., 2015; Teasdale et al., 2002). This decoupling is one of the proposed mechanisms by which contemplative training reduces emotional reactivity and broadens the cognitive-affective response (Garland et al., 2015). It is considered, in integrative neurocognitive models of contemplative transformation, as a marker of metacognitive maturation—a specific form of self-regulation distinct from direct emotion regulation (Vago and Silbersweig, 2012). The structural decoupling observed in G1 thus suggests that POEBRA produces precisely the kind of metacognitive awareness that other body-centered contemplative pedagogies have been theorized to support, with empirical evidence at the multivariate-architectural level rather than at the level of individual outcomes. By strengthening the connection between bodily sensations, attention regulation, and emotional awareness, POEBRA appears to help participants anchor their self-perception in lived bodily experience. This process of embodiment could explain the parallel evolution of self-esteem and anxiety reduction: as participants learn to recognize and trust their internal signals, they develop a more stable and benevolent relationship with themselves, promoting self-regulation and psychological integration.

The homogeneity of the between-group effect across the 20–70 age range of the sample, documented in Supplementary material S3, carries an additional implication for the interpretation of what is at work. Many psychological interventions show age-related sensitivity, with younger or older adults responding differentially depending on the developmental nature of the targeted capacity. The fact that the POEBRA effect is homogeneous across this five-decade age span suggests that what the program teaches is a learnable skill—one that adults of any age can acquire through structured practice—rather than a capacity tied to a particular stage of biological or cognitive development. This characteristic—combined with the multivariate architectural reorganization discussed above—invites further work on the scalability of POEBRA as a non-clinical intervention: the program appears deployable to a broad adult audience without need for stage-specific adaptation, while leaving open the empirical question of how the intensity and duration of the structured framework might be adjusted to sub-populations with specific needs.

## Limitations

Several limitations should be acknowledged. All outcome measures relied on self-report questionnaires, which, while validated, remain subject to social desirability and introspective biases. In addition, the screening for psychiatric or neurological conditions and psychotropic medication relied on participant self-report through the online enrolment questionnaire and was not corroborated by a standardized clinical evaluation. Further research combining self-reported measures with physiological data could bring a new perspective to the understanding of the embodied processes of change. We also believe that cross-referencing these data with the information of the experience logs, should confirm or mitigate those biases. The sample was predominantly female and composed of volunteers, which may limit generalizability. Moreover, the entirely online context, while advantageous for standardization, may have influenced participant engagement in ways that differ from face-to-face sessions. And like any structured group intervention, POEBRA inherently includes non-specific factors—instructor presence, social contact within the group, regular weekly engagement, and expectancy effects associated with participation in a named program—that the present design cannot separate from the specific contribution of its meditative and embodied content. Although matched active comparators have been developed to explore this issue in the mindfulness-based interventions literature (e.g., the Health Enhancement Program for MBSR; MacCoon et al., 2012), these designs also present methodological challenges, including imperfect matching of teacher engagement and expectancy effects (Davidson and Kaszniak, 2015). The present 2-arm design—comparing two forms of meditation practice that differ in the presence or absence of a structured and stepwise pedagogy—was therefore chosen to remain within a coherent meditative framework, despite the inherent limitations entailed. Further studies would be valuable to explore which specific components of POEBRA promote the development of an embodied sense of self as a central factor in psychoaffective regulation.

One methodological limitation specific to the inferential analyses should also be acknowledged. The Holm-Bonferroni correction was applied within each of three pre-specified families of tests rather than across all 18 tests, although the magnitude of the between-group effects (Cohen's |*d*| ≥ 0.44 for all six contrasts) makes the conclusions unlikely to be sensitive to this choice.

## Conclusion

Beyond the demonstration of clinical efficacy, these findings invite a broader reflection on the processes of contemplative transformation. POEBRA does not simply reduce anxiety or increase positive emotions; it promotes a reorganization of the self through embodied coherence, where perception, emotion, and cognition converge into an integrated experience of presence. The convergence of improvements across multiple dimensions supports the idea that meditation, when grounded in the body and accompanied by reflective guidance, can facilitate psychoaffective integration rather than isolated symptom relief.

In the end, POEBRA appears to offer a coherent and effective framework for developing embodied self-regulation and emotional resilience. The multivariate analyses further suggest that the program acts not only by amplifying the magnitude of psychoaffective improvement but by reorganizing the inner architecture of the response, with a distinctive decoupling of mood from the cognitive-evaluative dimension that is consistent with metacognitive maturation. The age-invariance of the between-group effect supports the deployability of the program to a broad adult audience. By combining sensorial exploration, metacognitive reflection, and relational support, it helps individuals transform self-critical or anxious patterns into a more compassionate and grounded sense of self. These findings support the development of body-based contemplative pedagogies that integrate emotional, cognitive, and somatic dimensions as a pathway toward psychological balance and wellbeing.

## Data Availability

The datasets presented in this study can be found in online repositories. The names of the repository/repositories and accession number(s) can be found below: https://doi.org/10.5281/zenodo.20312672.
