# Supplementary material for: Learning Full-Presence meditation using POEBRA: impacts on self-esteeem, benevolence, and anxiety
Source: Front Psychol. 2026 Jul 2;17:1833806. doi: 10.3389/fpsyg.2026.1833806 (PMC13372651; doi:10.3389/fpsyg.2026.1833806)
Supplement: Supplementary file 1 [file Data_Sheet_1.pdf]

Supplementary Material

The following information is complementary to the article entitled “Learning Full-Presence meditation using POEBRA: impacts on self-esteeem, benevolence and anxiety” (Anne Lieutaud & Helene Bourhis, 2026).

All short references mentioned here are detailed in the references of the article.

Table of contents

S1. - Example of a Sensorial Introspection-guided full-presence meditation sequence 2

S1.1 - The eleven-step standard protocol (Bois, 2018) 2
S1.2 - Synoptic mapping onto cognitive, metacognitive and psychoaffective spheres 3

S2. - Multivariate structure of the W8−W0 changes (Principal Component Analysis) 4

S2.1 - All-sample PCA (n = 125, group as supplementary qualitative variable) 4
S2.2 - Per-group PCAs (G1 and G2 separately) 5
S2.3 - Convergence of the all-sample and per-group analyses 8

S3. - Age-sensitivity analyses 9

S3.1 - Sensitivity ANCOVA with age as additional covariate. 9
S3.2 - Exploratory group × age interaction 9
S3.3 - Partial Spearman correlations between Δ outcomes and age, controlling for W0 and group 10

S4. - Complete statistics for the four families of tests underlying Tables 2 and 3 (raw and Holm-adjusted p-values) 10

S4.1 - Family F1: Within-group changes in G1 (n = 61) 11
S4.2 - Family F2: Within-group changes in G2 (n = 64) 11
S4.3 - Family F3: Between-group ANCOVA contrasts on (W8−W0), n = 125 11
S4.4 - Family F4: Spearman correlations between Δ outcomes within G1 12
S4.5 - Family F4: Spearman correlations between Δ outcomes within G2 12

S1. - Example of a Sensorial Introspection-guided full-presence meditation sequence

Because POEBRA is a structured pedagogical progression of 17 to 18 guided meditation sessions across an 8-week curriculum, the cognitive/metacognitive/psychoaffective mobilization evolves stage by stage, making a single emblematic transcript possibly misleading. We therefore document here the standard protocol of Sensorial Introspection developed by D. Bois — the invariant architectural backbone underlying all POEBRA Full-Presence Meditation sessions — together with a synoptic mapping onto the three spheres at play.

S1.1 - The eleven-step standard protocol (Bois, 2018)

The guiding instructions' chronological sequence can be summarized as follows:

1. Settle into a still posture in a seated position of one's choice and relax into the posture;
2. Establish the conditions of silence and close the eyes;
3. Listen to the silence, to the ambient sound atmosphere – therefore of the room and then of the group –, silence then becomes a presence as the qualitative dimension of the silence begins to color the sound atmosphere;
4. Turn inwards and perceive one's spatial position within the group and the room: in the center, to the right, to the left, in front, in the middle, at the back;
5. Then connect to the posture of the body, by observing its physical position and its tonic state;
6. Evaluate body states: perceive areas of tension and how they relax, maintain the muscular release;
7. Draw attention to the progression since the beginning of the session, starting from the physical to the psychical: perceiving oneself relaxed or tight, calm or tense, tranquil or anxious, serene or preoccupied. The participants are asked to evaluate their state by contrast with the beginning of the practice;
8. Through the eyelids, perception or not of a colored atmosphere (black, nothing or what colors) and its possible animation;
9. Take notice of the presence or not of a thought that emerges. Evaluate whether this thought is related to what is being experienced or not. The probing follows a sensorial progression: to perceive oneself or not in the process of thinking, and if yes evaluate what nature of thought this is, for example whether it is connected or not with the unfolding experience;
10. Identify the effects on the heart, alterity and openness to others, states such as softness, happiness and joy;
11. End with a breathing exercise to come back to daily life, in conjunction with a physical movement of the spine on inhalation and exhalation (slight extension of the spine on inhale and then slight flexion on exhale). Return to the neutral position and observe the end state.

This sequence is called a "standard protocol", as it is the kind of protocol used with beginners or at the beginning of a workshop. The instructions are neither suggestive nor inductive. When meditators are invited to observe their perceptions without judgment, it is always done by offering alternatives with reference to the "informative directivity" guidance principles (Bourhis H. (2008) La directivité informative dans le guidage d'une mise en sens de la subjectivité corporelle: une méthode pour mettre en évidence la donation de sens du corps sensible, *in « Sujet sensible et renouvellement du moi »,* Eds Bois, D., Josso, M.-C., & Humpich, M. Point d'appui). For example: for the sound atmosphere, "is it noisy or silent?"; for the visual sense, "does the luminosity appear outside or inside the body?"; for the proprioceptive sense, "is the posture tense or relaxed?" These instructions are

there to help the meditators discriminate their perceptions in real time, without the intervention of voluntary mental processes.

S1.2 - Synoptic mapping onto cognitive, metacognitive and psychoaffective spheres

Steps 1–3 (preparatory) and step 11 (closing) frame the practice through embodied settling and integrative return; they are not assigned to a single sphere but support the overall perceptual containment from which the three spheres can be mobilized.

Cognitive sphere – primarily steps 4 (spatial awareness within the group), 5 (postural and tonic-state perception), 6 (bodily evaluation: tension and release), 7 (attention to the physical-to-psychical progression), and 8 (perception or absence of a colored atmosphere through the eyelids). The cognitive engagement here is perceptual and discriminative: the practitioner sustains attention on body-anchored content and on contrasts (calm/tense, relaxed/tight) without elaborative judgment.

Metacognitive sphere – primarily step 9 (noticing the presence or absence of an emerging thought; evaluating whether the thought is connected to the unfolding experience; "perceiving oneself in the process of thinking"). The metacognitive engagement is the practitioner's capacity to observe the act of thinking itself, including its nature and its relevance to the present perceptual flow, without identification with the thought-content.

Psycho-affective sphere – primarily steps 7 (felt-quality of states: tranquil/anxious, serene/preoccupied), 10 (effects on the heart, alterity, openness, states such as softness, happiness, joy), and the final observation in step 11 (end-state). The psycho-affective engagement is the felt quality of the experience itself – an internal weather of states that emerges from sustained perceptual presence and is acknowledged without active modulation.

Across the eleven steps, the instructional style – non-suggestive, non-inductive, articulated through paired alternatives ("noisy or silent?", "tense or relaxed?", "outside or inside the body?") – is preserved unchanged throughout the POEBRA progression, even as the thematic accents of successive sessions evolve.

S2. - Multivariate structure of the W8−W0 changes (Principal Component Analysis)

This supplement reports the principal component analysis (PCA) of the W8 − W0 changes for the primary outcome (STAI-Y2, trait anxiety) and the five secondary outcomes (RSES, SCS, MAIA-2, PANAS-PA, PANAS-NA), conducted as complement to the univariate ANCOVA and bivariate Spearman analyses reported in the main text. The supplement is organized in two sections: S2.1 reports the principal analysis on the full sample (n = 125) with the group factor projected as a supplementary qualitative variable; S2.2 reports two complementary analyses computed separately within each group (n = 61 in G1, n = 64 in G2), with a focus on structural features that are specific to one group and would be lost in the all-sample view. They are followed by a overall comparative analysis in S2.3.

S2.1 - All-sample PCA (n = 125, group as supplementary qualitative variable)

A standard PCA was performed on the centered and scaled six-variable matrix of W8−W0 changes for the 125 participants. The group factor (G1 / G2) was projected on the resulting factorial space as a supplementary qualitative variable; that is, it did not contribute to the construction of the components but was used to label and to compute group-level confidence ellipses on the individual plots. The biplot reproduced as Figure 2 of the article is the central deliverable of this analysis. The numerical results are reported in the consolidated outputs S1_PCA_results.xlsx and summarized in the tables below.

Table S2.1.1. Eigenvalues, percentage of variance and cumulative percentage of variance (full sample, n = 125, 6 axes)

Axis	Eigenvalue	% variance	% cumulative
Dim.1	3.391	56.52	56.52
Dim.2	0.858	14.31	70.83
Dim.3	0.668	11.13	81.96
Dim.4	0.475	7.92	89.88
Dim.5	0.380	6.34	96.22
Dim.6	0.227	3.78	100.00

Note. The first three principal components account for 81.96 % of the total variance. The 'elbow' criterion and the cumulative variance suggest that the first three axes are amply sufficient to summarize the multivariate structure; subsequent axes are reported here for completeness but are not interpreted in the article.

Figure S2.1.1. Scree plot (full sample)

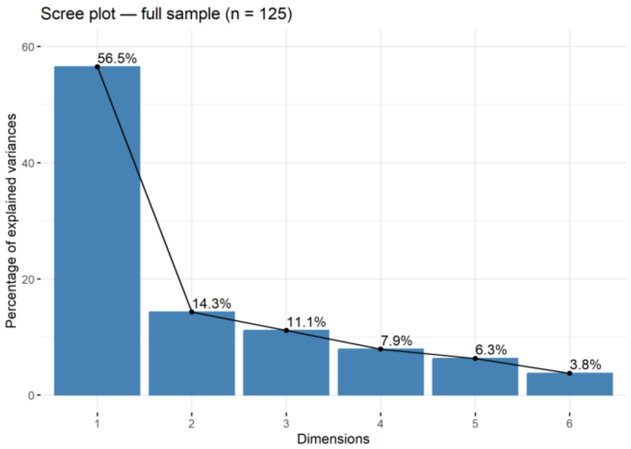

Note. Bar height = percentage of variance explained by each principal component. The decay after PC3 supports retaining 3 axes for interpretation.

Table S2.1.2. Variable coordinates, contributions and cos² (quality of representation) on PC1, PC2, PC3 (full sample)

	PC1			PC2			PC3		
Outcome (Δ)	*Coord*	*Contrib*	*cos²*	*Coord*	*Contrib*	*cos²*	*Coord*	*Contrib*	*cos²*
STAI-Y2	−0.902	24.02	0.814	+0.007	0.01	0.000	+0.111	1.86	0.012
RSES	+0.801	18.93	0.642	−0.124	1.79	0.015	−0.351	18.46	0.123
SCS	+0.838	20.72	0.703	+0.003	0.00	0.000	−0.097	1.42	0.010
MAIA-2	+0.635	11.91	0.404	+0.629	46.06	0.395	−0.124	2.29	0.015
PANAS-PA	+0.663	12.97	0.440	+0.162	3.08	0.026	+0.702	73.68	0.492
PANAS-NA	−0.623	11.46	0.389	+0.649	49.08	0.421	−0.124	2.29	0.015

Note. Coordinates indicate the projection of each variable on the principal components (= loadings scaled by the square root of the eigenvalue). On PC1 all six outcomes load strongly in the direction associated with clinical improvement: trait anxiety and negative affect load negatively, self-esteem, self-compassion, interoceptive awareness and positive affect load positively.

Contributions sum to 100 % within each axis. PC1 contributions are well-distributed across all six outcomes (between 11.5 % and 24.0 %), consistent with the unified-improvement interpretation. PC2 is dominated by MAIA-2 and PANAS-NA (≈ 95 % combined). PC3 is dominated by PANAS-PA (≈ 74 %).

Cos² indicates the quality with which each variable is represented on a given axis (squared cosine of the angle between the variable and the axis). Values close to 1 indicate that the variable lies nearly in the direction of the axis; values close to 0 indicate that the variable is poorly represented on that axis. STAI-Y2, RSES and SCS are predominantly represented on PC1; MAIA-2 and PANAS-NA are jointly represented on PC1 and PC2; PANAS-PA is jointly represented on PC1 and PC3.

Interpretation of the all-sample PCA

The first principal component (56.52 % of the variance) summarizes a unified pattern of clinical improvement across the six outcomes: trait anxiety and negative affect decrease (negative loadings) while self-esteem, self-compassion, interoceptive awareness and positive affect increase (positive loadings). The contributions to this axis are well-distributed across the six variables (11.5 % to 24.0 %), so PC1 is not driven by any single outcome but reflects a coordinated multivariate change. The second component (14.31 %) primarily contrasts changes in interoceptive awareness (MAIA-2) and in negative affect (PANAS-NA) with the rest of the outcomes; jointly these two variables account for ≈ 95 % of PC2's variance. The third component (11.13 %) is dominated by changes in positive affect (PANAS-PA: contribution ≈ 74 %), consistent with PANAS-PA carrying a behavioral-action dimension partially independent from the other five outcomes.

Projected on this factorial space, group G1 and group G2 differ primarily on PC1: the group centroids are clearly separated along the unified-improvement axis, with G1 displaced further in the improvement direction; the 95 % confidence ellipses on PC1 show limited overlap. This visual result aligns with the univariate ANCOVA contrasts reported in Table 2 of the main text and complements them by showing that the group effect operates on a single integrative dimension rather than on six disjoint outcomes.

S2.2 - Per-group PCAs (G1 and G2 separately)

To complement the all-sample analysis and to verify that the latent structure of the W8 − W0 changes is consistent across the two groups, two additional PCAs were computed separately on the G1 sub-sample (n = 61) and on the G2 sub-sample (n = 64). These analyses use the same six variables (W8−W0 changes on STAI-Y2, RSES, SCS, MAIA-2, PANAS-PA and PANAS-NA) and the same

standardization as the all-sample PCA. They allow us to inspect group-specific features of the latent structure that would be diluted in the pooled view.

S2.2a - PCA on G1 (n = 61)

Table S2.2a-1 — Eigenvalues (G1)

Axis	*Eigenvalue*	*% variance*	*% cumulative*
Dim.1	2.957	49.28	49.28
Dim.2	1.107	18.45	67.73
Dim.3	0.758	12.64	80.37
Dim.4	0.514	8.57	88.94
Dim.5	0.405	6.75	95.68
Dim.6	0.259	4.32	100.00

Table S2.2a-2 — Variable coordinates, contributions and cos² (first three axes, G1)

	PC1			PC2			PC3		
Outcome (Δ)	*Coord*	*Contrib*	*cos²*	*Coord*	*Contrib*	*cos²*	*Coord*	*Contrib*	*cos²*
STAI-Y2	−0.894	27.04	0.800	−0.056	0.28	0.003	−0.101	1.36	0.010
RSES	+0.793	21.26	0.629	−0.171	2.64	0.029	+0.348	15.94	0.121
SCS	+0.783	20.72	0.613	−0.036	0.12	0.001	+0.355	16.59	0.126
MAIA-2	+0.428	6.19	0.183	+0.798	57.55	0.637	−0.110	1.58	0.012
PANAS-PA	+0.676	15.45	0.457	+0.161	2.35	0.026	−0.583	44.78	0.339
PANAS-NA	−0.525	9.34	0.276	+0.641	37.06	0.410	+0.387	19.75	0.150

Note. Contributions in %.

Figure S2.2a-1 — Variables on axes 1-2, G1 (n = 61)

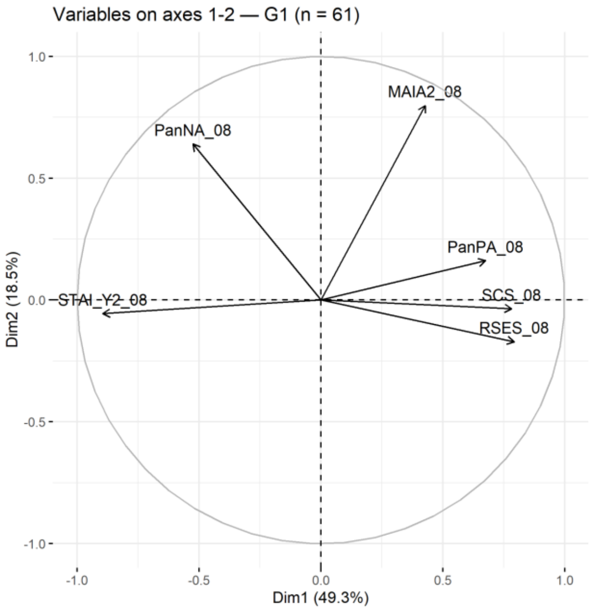

<u>Interpretation (G1)</u>

In G1, the first axis (PC1, 49.28 %) is dominated by trait anxiety (STAI-Y2 contribution = 27.04 %), self-esteem (RSES = 21.26 %), and self-compassion (SCS = 20.72 %), all loaded in the direction of clinical improvement. The second axis (PC2, 18.45 %) is essentially carried by interoceptive

awareness (MAIA-2 contribution = 57.55 %, cos² = 0.637) and negative affect (PANAS-NA = 37.06 %), which jointly account for ≈ 95 % of the variance on this axis. The third axis (PC3, 12.64 %) is dominated by positive affect (PANAS-PA contribution = 44.78 %, cos² = 0.339), with PANAS-PA negatively associated on this axis with self-esteem and self-compassion. This third axis can be read as expressing a behavioral-action dimension specific to the POEBRA program — a dimension of putting oneself into action through positive engagement — that operates partly independently of the more introspective components carried by the other variables.

S2.2b - PCA on G2 (n = 64)

Table S2.2b-1 — Eigenvalues (G2)

Axis	*Eigenvalue*	*% variance*	*% cumulative*
Dim.1	3.161	52.68	52.68
Dim.2	0.858	14.29	66.98
Dim.3	0.764	12.74	79.72
Dim.4	0.520	8.67	88.39
Dim.5	0.417	6.95	95.34
Dim.6	0.280	4.66	100.00

Table S2.2b-2 — Variable coordinates, contributions and cos² (first three axes, G2)

			PC1			*PC2*			*PC3*
Outcome (Δ)	*Coord*	*Contrib*	*cos²*	*Coord*	*Contrib*	*cos²*	*Coord*	*Contrib*	*cos²*
STAI-Y2	−0.873	24.10	0.762	+0.126	1.85	0.016	+0.054	0.38	0.003
RSES	+0.729	16.80	0.531	−0.376	16.52	0.142	+0.085	0.95	0.007
SCS	+0.830	21.80	0.689	+0.091	0.96	0.008	−0.003	0.00	0.000
MAIA-2	+0.649	13.34	0.422	+0.073	0.62	0.005	+0.676	59.70	0.456
PANAS-PA	+0.514	8.35	0.264	+0.801	74.75	0.641	−0.168	3.69	0.028
PANAS-NA	−0.702	15.61	0.493	+0.213	5.30	0.045	+0.519	35.28	0.270

Note. Contributions in %.

Figure S2.2b-1 — Variables on axes 1-2, G2 (n = 64)

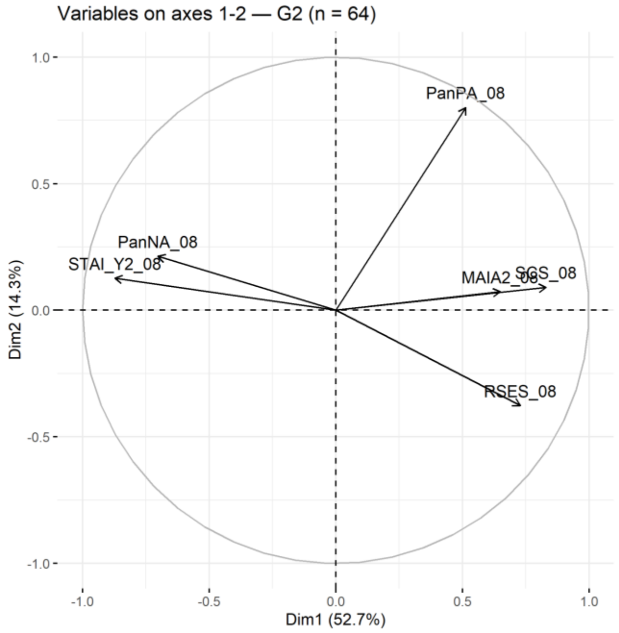

Interpretation (G2)

In G2, the first axis (PC1, 52.68 %) shows the same dominant unified-improvement structure as in G1 (and as in the all-sample analysis): all six outcomes load in the direction of clinical improvement, with contributions distributed across all six variables (8.35 % to 24.10 %). The second axis (PC2, 14.29 %) is qualitatively different from G1: it is essentially driven by changes in positive affect (PANAS-PA contribution = 74.75 %, $\cos^2 = 0.641$), with self-esteem (RSES) loading negatively on this axis (contribution = 16.52 %). PANAS-PA dominates PC2 in G2 in a way that has no counterpart in G1.

This atypical role of PANAS-PA in G2 reflects the substantial inter-individual heterogeneity of the positive-affect response to the G2 condition. Quantitatively, the coefficient of variation (standard deviation divided by the mean of the W8 − W0 change) of PANAS-PA in G2 is approximately 14 times that of PANAS-PA in G1: in G1 the change is large and consistently positive across participants, whereas in G2 the change has a small mean but a much greater dispersion, with some participants improving markedly while others do not improve or even decline. This polarization of the positive-affect response – visible as the dominant role of PANAS-PA on PC2 of the G2-only PCA – is consistent with the interpretation that the G2 condition produced a more differentiated reactivity of positive moods, with a subset of participants benefiting substantially and others not. The third axis (PC3, 12.74 %) is dominated by interoceptive awareness (MAIA-2 contribution = 59.70 %, $\cos^2 = 0.456$) and to a lesser extent by negative affect (PANAS-NA = 35.28%).

S2.3 - Convergence of the all-sample and per-group analyses

The two analytical perspectives converge on the same overall conclusion. (i) On the dominant axis of variance (PC1), all six outcomes load in the direction of clinical improvement, with well-distributed contributions; this structure is observed in the all-sample analysis as well as in each of the per-group analyses, supporting the interpretation that the eight-week program produces a coordinated multivariate transformation rather than independent effects on disjoint constructs. (ii) The all-sample biplot (Figure 2 of the article) shows that participants of group G1 are projected substantially further than participants of group G2 along the unified-improvement axis, in concordance with the univariate ANCOVA contrasts reported in Table 2 of the main text. (iii) The per-group analyses reveal one notable group-specific feature: in G2, the second principal component is dominated by the heterogeneity of the positive-affect response (PANAS-PA), a feature that has no counterpart in G1 and that points to a more differentiated inter-individual reactivity to the G2 condition on the affective register.

S3. - Age-sensitivity analyses

In order to verify that the primary group effect on trait anxiety, and the concordant effects on the five secondary outcomes (RSES, SCS, MAIA-2, PANAS-PA, PANAS-NA), are not confounded by participants' age, we conducted a three-component sensitivity package on the same dataset (n=125). All analyses are complementary to the primary ANCOVA reported in Table 2 of the main text. They are reported as sensitivity / exploratory analyses with α = .05 (no further multiplicity adjustment beyond the primary Holm-Bonferroni applied to F3).

S3.1 - Sensitivity ANCOVA with age as additional covariate.

For each of the six outcomes, the ANCOVA model (W8−W0) ~ W0 + group was extended to (W8−W0) ~ W0 + group + age, and the estimated marginal mean (EMM) difference between groups was re-estimated in order to verify that the conclusions of the primary analysis are not modified by adjustment for age.

	Age adjusted Ancova				*Primary Ancova (no age)*		
Outcome (Δ)	*EMM diff [95% CI]*	*p (group age)*	*Age slope (β)*	*P (age)*	*EMM diff [95% CI]*	*p (Holm)*	*n*
STAI-Y2 (Δ)	-8.170 [-10.764; -5.577]	< .001	+0.0690	.2455	-8.070 [-10.660; -5.480]	< .001	125
RSES (Δ)	+3.371 [+2.054; +4.688]	< .001	-0.0036	.9044	+3.370 [+2.060; +4.670]	< .001	125
SCS (Δ)	+0.553 [+0.374; +0.732]	< .001	-0.0086	.0372	+0.540 [+0.360; +0.720]	< .001	125
MAIA-2 (Δ)	+0.670 [+0.454; +0.886]	< .001	-0.0063	.1907	+0.660 [+0.450; +0.880]	< .001	125
PANAS-PA (Δ)	+4.116 [+2.354; +5.878]	< .001	-0.0052	.8981	+4.110 [+2.360; +5.860]	< .001	125
PANAS-NA (Δ)	-3.215 [-5.270; -1.160]	.0024	-0.0820	.0851	-3.330 [-5.400; -1.260]	.0018	125

Note. Age slope β = unstandardized regression coefficient of age (years) in the age-adjusted ANCOVA; negative values indicate slightly less change per additional year of age. p (age) and p (group age) are raw two-sided p-values from the age-adjusted ANCOVA. The right-hand columns reproduce the primary Table 2 ANCOVA estimates (without age) for direct side-by-side comparison: EMM differences and 95% CIs shift by ≤ 0.10 unit on each scale, and Holm-significance is preserved on the primary outcome and on the five secondary outcomes.

S3.2 - Exploratory group × age interaction

For each outcome, the model (W8−W0) ~ W0 + group + age was compared to the augmented model (W8−W0) ~ W0 + group * age via a likelihood-ratio F-test on the group × age interaction term. The objective is to document that no quantitatively meaningful effect modification by age is present.

Outcome (Δ)	*F (group × age)*	*df_1*	*df_2*	*p (interaction)*	*Conclusion (α = .05)*	*n*
STAI-Y2 (Δ)	0.0204	1	120	.8866	ns	125
RSES (Δ)	0.0681	1	120	.7946	ns	125
SCS (Δ)	0.1333	1	120	.7157	ns	125
MAIA-2 (Δ)	0.4014	1	120	.5276	ns	125
PANAS-PA (Δ)	0.2333	1	120	.63	ns	125
PANAS-NA (Δ)	0.4668	1	120	.4958	ns	125

Note. None of the six interaction tests is statistically significant at α = .05 (smallest p = .496). The magnitude of the group effect on W8 − W0 changes is therefore homogeneous across the age range of the sample, both for the primary outcome (STAI-Y2) and for the five secondary outcomes. ns = no significant interaction

S3.3 - Partial Spearman correlations between Δ outcomes and age, controlling for W0 and group

For each outcome, the partial Spearman correlation between the W8 − W0 change and participants' age was computed, holding constant the W0 baseline and group assignment. The objective is to confirm absence of a substantive monotonic age–response association beyond what is captured by the linear age covariate of S3.1.

Outcome (Δ)	*Partial Spearman ρ (Δ, age | W0, group)*	*p (raw)*	*Conclusion (α = .05)*	*n*
STAI-Y2 (Δ)	+0.0672	.4602	ns	125
RSES (Δ)	-0.0142	.8761	ns	125
SCS (Δ)	-0.1719	.0572	ns	125
MAIA-2 (Δ)	-0.1302	.1511	ns	125
PANAS-PA (Δ)	-0.0185	.8394	ns	125
PANAS-NA (Δ)	-0.1430	.1147	ns	125

Note. Computed with the ppcor package in R. All six partial Spearman ρ are small in magnitude (|ρ| ≤ 0.18, smallest p = .057 for SCS, all others p > .11) and none reaches statistical significance at α = .05. ns = no significant interaction.

<u>Conclusion of the age-sensitivity package</u>

The three sensitivity analyses converge on a single conclusion: the primary ANCOVA estimate of the between-group effect on trait anxiety, and the concordant estimates on the five secondary outcomes, are robust to age adjustment. (i) The age-adjusted EMM differences (S3.1.) deviate from the primary estimates by at most a hundredth of a unit on each scale and remain Holm-significant on all six outcomes. (ii) No group × age interaction approaches significance (S3.2.; smallest p = .496). (iii) No substantive monotonic age–response association exists once W0 and group are controlled for (S3.3.; |ρ| ≤ 0.18, all p > .057). Reported as sensitivity / exploratory analyses, no formal claim is made beyond robustness of the primary conclusions to age adjustment.

S4. - Complete statistics for the four families of tests underlying Tables 2 and 3 (raw and Holm-adjusted p-values)

This supplement provides the complete numerical output of the four statistical families summarized in Tables 2 and 3 of the main text. The trial reports one primary outcome (STAI-Y2 trait anxiety) and five secondary outcomes (RSES, SCS, MAIA-2, PANAS-PA, PANAS-NA); both raw p-values and Holm-Bonferroni–adjusted p-values are reported, alongside the test statistic and degrees of freedom, so that the reader can verify the multiplicity correction.

<u>Test family definitions</u>: F1 = within-G1 changes (W8−W0), 6 paired t-tests; F2 = within-G2 changes (W8−W0), 6 paired t-tests; F3 = between-group ANCOVA contrasts on (W8−W0), 6 EMM differences with 95% CI; F4 = Spearman rank-order correlations between Δ outcomes within each group, 15 pairs per group. The Holm-Bonferroni step-down correction was applied within each family (α = .05 family-wise).

S4.1 - Family F1: Within-group changes in G1 (n = 61)

Table F1: one-sample t-tests on the W8−W0 difference within group G1, 6 outcomes, Holm-Bonferroni correction within the family.

Outcome (Δ)	*Mean (SD)*	*t*	*df*	*p (raw)*	*p (Holm)*	*Cohen's d*
STAI-Y2 (Δ)	-10.459 (8.447)	-9.671	60	< .001	< .001	-1.238
RSES (Δ)	+4.410 (4.216)	+8.168	60	< .001	< .001	+1.046
SCS (Δ)	+0.785 (0.602)	+10.185	60	< .001	< .001	+1.304
MAIA-2 (Δ)	+0.888 (0.655)	+10.590	60	< .001	< .001	+1.356
PANAS-PA (Δ)	+4.459 (5.638)	+6.177	60	< .001	< .001	+0.791
PANAS-NA (Δ)	-7.000 (7.301)	-7.489	60	< .001	< .001	-0.959

S4.2 - Family F2: Within-group changes in G2 (n = 64)

Table F2: one-sample t-tests on the W8−W0 difference within group G2, 6 outcomes, Holm-Bonferroni correction within the family.

Outcome (Δ)	*Mean (SD)*	*t*	*df*	*p (raw)*	*p (Holm)*	*Cohen's d*
STAI-Y2 (Δ,)	-2.344 (6.899)	-2.718	63	.0085	.0254	-0.340
RSES (Δ)	+0.844 (3.872)	+1.743	63	.0861	.1723	+0.218
SCS (Δ)	+0.260 (0.548)	+3.799	63	< .001	.0013	+0.475
MAIA-2 (Δ)	+0.350 (0.639)	+4.386	63	< .001	< .001	+0.548
PANAS-PA (Δ)	+0.344 (6.193)	+0.444	63	.6585	.6585	+0.056
PANAS-NA (Δ)	-3.844 (6.936)	-4.434	63	< .001	< .001	-0.554

S4.3 - Family F3: Between-group ANCOVA contrasts on (W8−W0), n = 125

Table F3: ANCOVA modelling (W8−W0) ~ W0 + group, on 125 participants.

Outcome (Δ)	*EMM diff [95% CI]*	*t*	*df*	*p (raw)*	*p (Holm)*	*Cohen's d*	*Slope p (W0 × group)*
STAI-Y2 (Δ)	-8.068 [-10.660; -5.476]	-6.163	122	< .001	< .001	-1.055	0.209
RSES (Δ)	+3.365 [+2.057; +4.674]	+5.090	122	< .001	< .001	+0.882	0.044
SCS (Δ)	+0.541 [+0.360; +0.722]	+5.918	122	< .001	< .001	+0.912	0.676
MAIA-2 (Δ)	+0.661 [+0.445; +0.877]	+6.060	122	< .001	< .001	+0.831	0.007
PANAS-PA (Δ)	+4.109 [+2.358; +5.860]	+4.646	122	< .001	< .001	+0.694	0.774
PANAS-NA (Δ)	-3.331 [-5.398; -1.263]	-3.189	122	.0018	.0018	-0.444	0.284

*The group effect is reported as the estimated marginal mean (EMM) difference G1 − G2 with 95% CI. Slope homogeneity p (W0 × group) = p-value of the W0 × group interaction term in the augmented non-parallel-slopes model (W8−W0) ~ W0 * group. The ANCOVA assumes equal slopes of the relationship between (W8−W0) and W0 across groups (homogeneity-of-slopes assumption). A value below .05 indicates that the slopes differ between groups: the parallel-slopes EMM estimate should then be interpreted with caution.*

S4.4 - Family F4: Spearman correlations between Δ outcomes within G1

Table F4-G1 (G1, n = 61, 15 pairs): Spearman rank-order correlations between W8−W0 changes for the 15 unique outcome pairs computed within group G1.

Outcome 1	*Outcome 2*	*Spearman ρ*	*p (raw)*	*p (Holm)*	*Sig. (Holm)*
STAI-Y2	*RSES*	-0.643	< .001	< .001	Yes
STAI-Y2	*SCS*	-0.680	< .001	< .001	Yes
STAI-Y2	*MAIA-2*	-0.321	.0118	.0824	No
STAI-Y2	*PANAS-PA*	-0.520	< .001	< .001	Yes
STAI-Y2	*PANAS-NA*	+0.366	.0037	.0408	Yes
RSES	*SCS*	+0.560	< .001	< .001	Yes
RSES	*MAIA-2*	+0.220	.0884	.305	No
RSES	*PANAS-PA*	+0.263	.0406	.2031	No
RSES	*PANAS-NA*	-0.351	.0056	.0561	No
SCS	*MAIA-2*	+0.229	.0763	.305	No
SCS	*PANAS-PA*	+0.339	.0075	.0597	No
SCS	*PANAS-NA*	-0.286	.0257	.154	No
MAIA-2	*PANAS-PA*	+0.222	.085	.305	No
MAIA-2	*PANAS-NA*	+0.088	.4998	.4998	No
PANAS-PA	*PANAS-NA*	-0.348	.0059	.0561	No

Holm-Bonferroni correction applied within the 15 tests.

S4.5 - Family F4: Spearman correlations between Δ outcomes within G2

Table F4-G2 (G2, n = 64, 15 pairs): Spearman rank-order correlations between W8−W0 changes for the 15 unique outcome pairs computed within group G2.

Outcome 1	*Outcome 2*	*Spearman ρ*	*p (raw)*	*p (Holm)*	*Sig. (Holm)*
STAI-Y2	*RSES*	-0.482	< .001	< .001	Yes
STAI-Y2	*SCS*	-0.644	< .001	< .001	Yes
STAI-Y2	*MAIA-2*	-0.282	.024	.096	No
STAI-Y2	*PANAS-PA*	-0.344	.0054	.0379	Yes
STAI-Y2	*PANAS-NA*	+0.567	< .001	< .001	Yes
RSES	*SCS*	+0.360	.0034	.0309	Yes
RSES	*MAIA-2*	+0.192	.1286	.3859	No
RSES	*PANAS-PA*	+0.166	.1888	.3859	No
RSES	*PANAS-NA*	-0.374	.0023	.0233	Yes
SCS	*MAIA-2*	+0.351	.0045	.0357	Yes
SCS	*PANAS-PA*	+0.386	.0017	.0182	Yes
SCS	*PANAS-NA*	-0.528	< .001	< .001	Yes
MAIA-2	*PANAS-PA*	+0.316	.0111	.0666	No
MAIA-2	*PANAS-NA*	-0.187	.1381	.3859	No
PANAS-PA	*PANAS-NA*	-0.313	.0118	.0666	No

Holm-Bonferroni correction applied within the 15 tests.
